# Editorial: Women’s nutrition and bariatric surgery

**DOI:** 10.3389/fendo.2025.1546308

**Published:** 2025-02-07

**Authors:** Violeta Moizé, Rosa Casas

**Affiliations:** ^1^ Endocrinology and Nutrition. Obesity Unit, Hospital Clinic Barcelona and Institut d’Investigacions Biomèdiques August Pi Sunyer (IDIBAPS), Barcelona, Spain; ^2^ Instituto de Salud Carlos III, Centro de Investigación Biomédica en Red de Diabetes y Enfermedades Metabólicas Asociadas (CIBERDEM), Madrid, Spain; ^3^ Centro de Investigación Biomédica en Red (CIBEROBN) de Fisiopatología de la Obesidad y Nutrición, Instituto de Salud Carlos III, Madrid, Spain; ^4^ Nutrition and Food Safety Research Institute (INSA), University of Barcelona, Santa Coloma de Gramanet, Spain; ^5^ Department of Internal Medicine, Hospital Clinic, Institut d’Investigació Biomèdica August Pi i Sunyer (IDIBAPS), University of Barcelona, Barcelona, Spain

**Keywords:** recurrent weight gain, sarcopenia, sleep apnea, dietary patterns, anemia, pregnancy, sexual function, body composition

Obesity is a progressive, chronic and stigmatizing disease characterized by abnormal or excessive accumulation of fat that can adversely affect health. With an estimated 4.7 million deaths worldwide, obesity is the fourth leading cause of premature death. Bariatric surgery (BS) is considered the most effective therapeutic intervention for achieving and maintaining long-term weight loss and improving obesity-related comorbidities (1). Research shows that women are more likely than men to seek clinical intervention (2), and the majority of patients undergoing bariatric surgery are women, according to recent global reports. Although the underlying causes of this gender disparity are unclear, it has been suggested that stigma, the prevalence of psychological problems and poor body image are more common in women than in men (3). Similarly, the outcomes of BS often differ between men and women. These differences are typically attributed to variations in hormonal profiles, body composition, and social and behavioral factors (4–6). Studies show that women tend to lose a higher percentage of excess weight compared to men (4). However, men may experience greater absolute weight loss due to their higher initial body weight (4, 5). Women are more likely to report greater improvements in quality of life post-surgery, while men may experience less psychological benefit despite similar physical health improvements (6). It has been suggested that men may find it difficult to consider BS due to a lack of knowledge about surgical complications and postoperative outcomes (7). Qualitative research has shown that after BS, men begin a complex and prolonged life-changing process involving both increased agency and health-related experiences. Much remains to be learned about the impact of gender as a biological variable but also as a behavioral and social variable. The analysis of gender differences in routinely performed procedures is therefore crucial, as data suggest the existence of a gender gap in the quality and experience of BS. As the causes and effects of these gender differences on long-term outcomes are not fully understood, it is important to study the outcomes of bariatric surgery by gender.

Evidence suggests that obesity has a negative impact on overall quality of life, particularly in women, as obesity is a cause of stigma, menstrual disorders, sexual dysfunction, infertility, changes in bone metabolism, urinary incontinence, sarcopenia, miscarriage, and psychological problems and increases the risk of almost all pregnancy complications (8). For these reasons, BS has become a popular approach to weight loss in women with obesity. This Research Topic has attracted papers that examine the unique nutritional challenges, health outcomes, and physiological changes faced by women undergoing BS, in addition to interventions and strategies to optimise their long-term health and quality of life. Furthermore, it forms a collection that expands our knowledge of the intersection of women’s nutrition and bariatric surgery, shedding light on gender-specific considerations, pre- and post-operative care, and the broader implications for women’s health and well-being in the context of surgical weight management.

One article in this Research Topic highlighted how BS improves glucose metabolism by examining the changes in hormones secreted by the three major metabolic tissues - pancreas, adipose tissue and gut - along with differences in inflammatory cytokines of multiple origins between the remission and non-remission groups. These findings were reported by Kim et al., and can be considered important data for obtaining non-invasive markers of obesity and metabolic syndrome.

A clearly important aspect of women’s health post BS is the impact of both obesity and its consequences on reproductive health. Women with obesity have higher rates of PCOS and irregular menses with reduced fertility. PCOS increases AMH levels, which is a controversial marker of ovarian reserve. Studies show a significant drop in anti-Müllerian hormone (AMH) levels shortly after BS, but the cause remains unclear. It is uncertain whether the drop in AMH indicates a true loss of ovarian reserve due to a reduction in follicle count, whether it is a temporary functional change in folliculogenesis that resolves after the peak weight loss phase, or whether it represents a “normalization” of the elevated levels observed in PCOS. Adipokines and certain nutritional parameters (e.g. Vitamin D, Vitamin B12, ferritin, prealbumin, total cholesterol, etc.) may also affect AMH levels. While antral follicular count (AFC) is an important non-hormonal indicator of ovarian reserve, there is limited data on how obesity affects this measure. Related to this, another article in this Research Topic by Andreu et al. observed a significant decrease in AMH levels and a decrease in AFC during the first 12 months of BS, with both markers stabilizing thereafter. AMH variations were associated with reproductive and metabolic parameters, particularly changes in androgen and insulin levels. It remains unclear whether these changes reflect an effect on follicular physiology and granulosa cell function or a true quantitative loss of ovarian follicles. Clinically, it is important to note that markers of ovarian reserve, such as AMH, indicate the number of oocytes in the ovaries at a given time and the response of the ovaries to *in vitro* fertilization treatments. Therefore, if the observed reduction in AMH after BS represents a real reduction in ovarian reserve, it may reflect a reduced response to ovarian  stimulation in assisted reproduction. It may not indicate any reduced overall  reproductive capacity or increase the risk of future infertility.

It is interesting to note that previous research has linked the presence of persistent organic pollutants (POPs) to obesity. Plasma levels of these pollutants have been found to correlate with BMI, gender and rapid weight loss. This suggests that there may be a transfer between the circulatory system and adipose tissue. There is also a lack of knowledge about how these compounds are affected by weight loss through bariatric surgery. Thus, although previous research has linked the presence of persistent organic pollutants (POPs) to obesity, little is known about how these compounds are affected by weight loss after bariatric surgery. For the first time in this Research Topic, Díaz-González et al., analyzed 353 compounds in the sera of 59 women with obesity before and after surgery and found a significant increase in both detection rates and concentrations of several pollutants, including circulating POPs, after rapid weight loss. In addition, gender differences were observed in the correlation between concentrations of naphthalene, phenanthrene, PCB-138, weight loss, total lipids and time after surgery. These compounds are often lipid-soluble and stored in adipose tissue. With any lipid mobilization (weight loss, lactation), these compounds mobilize into the serum. Therefore, the increased levels may be due to the depletion of adipose stores of the lifelong accumulation of these compounds for elimination. This was a well-known issue with lipid-soluble pesticides in Inuit who ate fish from rivers containing agricultural pesticides- it was recommended that these women not breastfeed to prevent infant exposure (9). These contaminants can cause endocrine, metabolic and molecular disruptions that can lead to the development of multiple pathologies, such as endometriosis, elevated inflammatory markers, cardiovascular disease, and prostate and breast cancer. In addition, some of the chemicals released during weight loss are thought to contribute to the development of cancer or neurotoxicity. This highlights the need for legislative and biomonitoring efforts to prevent the development of exposure-related diseases.

Gender is an important factor for research excellence and equity in health outcomes. Attention to gender differences and the integration of the gender dimension in research has been shown to improve the quality, rigour, reproducibility and creativity of research, to increase the societal relevance of research and innovation, and to promote greater application of its results to the population.

Differences in outcomes between men and women in BS have not been extensively studied. This Research Topic will highlight relevant scientific issues and make a significant contribution with new knowledge related to women’s nutrition and bariatric surgery. It also aims to encourage further research in this area to help healthcare providers individualise the best therapeutic approach for women and optimise health-related outcomes associated with bariatric surgery.
